# Evaluating physiological stress in Sumatran tigers (*Panthera tigris* ssp. *sumatrae*) managed in Australian zoos

**DOI:** 10.1093/conphys/cou038

**Published:** 2014-10-06

**Authors:** Tempe Parnell, Edward J. Narayan, Michael J. L. Magrath, Sheila Roe, Giles Clark, Vere Nicolson, Patrick Martin-Vegue, Al Mucci, Jean-Marc Hero

**Affiliations:** 1 Environmental Futures Research Institute, School of Environment, Griffith University, Gold Coast campus, Queensland 4222, Australia; 2 Wildlife Conservation and Science, Zoos Victoria, PO Box 74, Parkville, Victoria 3052, Australia; 3 Australia Zoo, Steve Irwin Way, Beerwah, Queensland 4519, Australia; 4 Dreamworld, Parkway Coomera, Queensland 4209, Australia

**Keywords:** Australia, faecal cortisol metabolites, stress, tigers, welfare, zoos

## Abstract

Glucocorticoid quantification using non-invasive methods provides a powerful tool for assessing the health and welfare of wildlife in zoo-based programmes. In this study, we provide baseline data on faecal-based glucocorticoid (cortisol) monitoring of Sumatran tigers (*Panthera tigris* ssp. *sumatrae*) managed at the Melbourne Zoo in Victoria, Australia. We sampled five tigers daily for 60 days. Faecal cortisol metabolites (FCMs) in tiger faecal extracts were quantified using enzyme immunoassays that were successfully validated using parallelism and accuracy recovery checks. Two female tigers had significantly higher mean FCM levels than the two males and another female, suggesting that females may have higher FCM levels. A significant elevation was noted in the FCM levels for one female 2 days after she was darted and anaesthetized; however, the FCM levels returned to baseline levels within 3 days after the event. Comparative analysis of FCM levels of tigers sampled at Melbourne Zoo with tigers sampled earlier at two other Australian Zoos (Dreamworld Themepark and Australia Zoo) showed that FCM levels varied between zoos. Differences in the enclosure characteristics, timing of sampling, size and composition of groupings and training procedures could all contribute to this variation. Overall, we recommend the use of non-invasive sampling for the assessment of adrenocortical activity of felids managed in zoos in Australia and internationally in order to improve the welfare of these charismatic big cats.

## Abstract (precis)

We have successfully employed non-invasive fecal based glucocorticoid monitoring to evaluate fecal cortisol metabolites in male and females Sumatran tigers from Australian Zoos. Our study provides baseline FCM data which will be crucial for evaluting the health and welfare of big cats in zoos around the world.

## Introduction

*Ex situ* management of wildlife through zoological programmes is of particular importance for endangered species at risk of extinction in their natural environment and whose existence relies on human intervention and support through captive management strategies (Hutchins *et al.*, 2003; Dehnhard *et al.*, 2008). Understanding the physiology of animals in captivity and how their health can be jeopardized by physiological stress from novel environments and husbandry practices are crucial for maintaining sustainable and healthy captive populations (Hing *et al.*, 2014). Conservation physiology is an emerging discipline of conservation biology (Cooke *et al.*, 2013), which applies non-invasive technologies to provide quantitative assessment of the various effects of environmental stress in wildlife (Wielebnowski *et al.*, 2002a, b; Narayan *et al.*, 2013a, b). Faecal cortisol metabolite (FCM) enzyme immunoassay (EIA) and radioimmunoassay are widely used to measure baseline levels of FCMs, and the magnitude of change (rise or fall) in FCM is a widely accepted index of physiological stress (Young *et al.*, 2004). Quantitative assessments of physiological stress can also support zoo programmes through improvements to husbandry practices so that the physiological adaptation and wellbeing of animals is enhanced and reproductive success achieved (Young *et al.*, 2004).

The wild population of the Sumatran tiger sub-species is exclusively found on the Indonesian island of Sumatra. With fewer than 400 individuals remaining in the wild, this sub-species is of particularly high priority for captive breeding programmes due to their critically endangered status (CR; IUCN Red List; Chundawat *et al.*, 2011). Acquiring new knowledge about tiger stress physiology will allow zoos to progress and excel in areas of husbandry practices, veterinary care, nutrition, exhibit designs and population genetics (Wielebnowski, 2003). Despite its usefulness and practicality, only limited research to date has focused on non-invasive methods for evaluating the stress physiology of tigers (Naidenko *et al.*, 2011; Narayan *et al.*, 2013a). Terio *et al.* (2004) highlighted the need for more data on baseline FCM concentrations for felids in captivity. Pride (2005) also suggested that identification of sub-clinical physiological stress using FCM indices can allow managers to focus on high-risk animals that are most vulnerable. We urgently require information on baseline FCM profiles of tigers obtained from as many zoos as possible to develop a comprehensive understanding of how captive tigers respond to different environmental and management interventions. This information will allow us to compare the stress hormone levels of tigers between zoo facilities, which will significantly improve our ability to identify, manage, minimize and mitigate threats to tigers in zoos. Narayan *et al.* (2013a) published the first detailed study on the stress physiology of tigers in captivity at two Australian zoos (Dreamworld Themepark and Australia Zoo), including laboratory and biological validation of faecal cortisol metabolite analysis for two tiger sub-species, the Bengal (*Parthera tigris tigris*) and Sumatran tigers (*Parthera tigris sumatrae*).

In this study, we aimed to quantify baseline FCM levels of Sumatran tigers managed at the Melbourne Zoo (Victoria, Australia). The main objectives were as follows: (i) to quantify and compare the range and magnitude of FCM levels between male and female Sumatran tigers; (ii) to examine the physiological response of individual tigers to specific stressors (darting and anaesthesia); and (iii) to compare the mean FCM levels of male and female Sumatran tigers at three Australian zoos (Melbourne Zoo, Australia Zoo and Dreamworld Themepark).

## Materials and methods

### Sample collection

We measured FCMs in five Sumatran tigers at Melbourne Zoo (Victoria, Australia) and compared these with FCM levels in Sumatran tigers from Dreamworld Themepark and Australia Zoo. Melbourne Zoo is managing these five Sumatran tigers as valuable members of Australia's national tiger breeding programme (Table 1). These tigers are related to each other, because four of the individuals (two male and two female) are siblings from the same litter, born to the fifth individual (mother, female). The study was designed to obtain a profile of FCMs by time (days) for each tiger. Faecal samples were collected daily when available for each tiger over 60 days, beginning in February 2013. All fresh samples (<12 h old) were collected early in the morning from the cage of individual tigers and preserved immediately by freezing at −20°C in sealed plastic bags.

**Table 1:** Descriptive statistics for faecal cortisol metabolite values of tigers (*n* = 5) at Melbourne Zoo, Victoria, Australia

Tiger	Name	Total sampling period (days)	Mean FCMs [ng (g dry faeces) ^−1^]	SEM	CV (%)	Minimum	Maximum
Male 1	Aceh	14	4.86	2.01	154	0.24	30.58
Male 2	Hutan	13	15.56	7.67	177	1.82	103.71
Female 1	Rani	32	48.43	7.26	154	4.66	179.49
Female 2	Indrah	21	15.31	3.87	115	2.59	67.59
Female 3	Binjai	20	43.60	11.63	119	5.75	215.31

Abbreviations: CV, coefficient of variation; FCMs, faecal cortisol metabolites.

### Extraction of FCMs

Extraction of FCMs followed methods previously described by Wielebnowski *et al.* (2002a) for the clouded leopard (*Neofelis nebulosa*) and used recently in numerous studies by our research group (Narayan *et al.*, 2012, 2013a, b, 2014; Evans *et al.*, 2013). Briefly, all faecal samples were dehydrated in a lyophilizer, then sieved and pulverized. Homogenized faecal powder (0.2 g) was boiled in a 90% ethanol solution for 20 min to achieve maximal binding of the hormone metabolites to the aqueous solution. The samples were centrifuged for a 5 min period at 6050 × *g*, allowing separation of any remaining solids. From this, the supernatant was recovered and taken to dryness in a fume cupboard. Extracted particles adhering to the vessel wall were reconstituted in an assay buffer (39 mm NaH_2_PO_4_.H_2_O, 61 mm NaHPO_4_, 15 mm NaCl and 0.1% bovine serum albumin, pH 7.0) in preparation for analysis of FCMs by EIA.

### Faecal cortisol metabolite EIA

Laboratory protocols followed those based on established work on the greater bilby (*Macrotis lagotis*) described by Narayan *et al.* (2012), the koala (*Phascolactus cinereus*) described by Narayan *et al.* (2013b) and, most recently, on the tiger (Narayan *et al.*, 2013a). This procedure involved the quantification of the FCM concentration of each sample through duplicate assays performed in 96-well Nunc MaxiSorp™ plates. Cortisol antisera and horseradish peroxidase were procured from the University of California, UC Davis. Concentrations were determined using a polyclonal anti-cortisol antiserum (R4866) diluted at 1:15 000, horseradish peroxidase-conjugated cortisol label diluted at 1:80 000 and cortisol standards (1.56–400 pg per well). Specificity of the R4866 anti-cortisol antiserum to the targeted cortisol antigen is reported at 100%, in comparison to 10% with other steroids tested (Narayan *et al.*, 2010).

Nunc MaxiSorp™ plates were coated with 50 μl of antibody diluted in a coating buffer (50 mmol^−1^ bicarbonate buffer, pH 9.6) to the appropriate concentration. Plates were then incubated for at least 12 h at 4°C before being washed using an automated plate washer supplied with phosphate-buffered saline containing 0.5 ml Tween 20 to rinse off all unbound antibody. Dilutions in assay buffer to the appropriate concentration were prepared for stocks of standards, high- and low-binding internal controls, faecal extracts and horseradish peroxidase labels. For all EIAs, 50 μl of standard, internal control and diluted faecal extract was added to each well. Then, 50 μl of the corresponding horseradish peroxidase label was added to each well. These plates were then incubated at room temperature for 2 h. Plates were washed again after incubation, and 50 μl of a substrate buffer (0.01% tetramethylbenzidine and 0.004% H_2_O_2_ in 0.1 m acetate citrate acid buffer, pH 6.0) was added to each well. Stopping solution (50 μl of 0.5 mol l^−1^ H_2_SO_4_) was added so that the optical density of the zero wells would read between 0.7 and 1.0, after ∼20 min incubation at room temperature. Plates were then read at 450 nm (reference 630 nm) on a microplate reader to determine the FCM concentration detected for each assay.

### Laboratory EIA validation

Methods necessary to validate the EIA methodology for measuring FCMs in the tiger required the following conditions to be met: (i) parallelism, i.e. parallel displacement of serial dilutions of the pooled tiger faecal extracts to the respective cortisol standard curve; and (ii) extraction efficiency, i.e. significant recovery of exogenous steroid standard added to faecal extracts.

### Statistical analysis

All hormone data are expressed as the FCM concentration (in nanograms per gram) on a dry faeces weight basis. As individuals were repeatedly sampled over time, time series analysis was employed. This enabled the construction of a unique profile for each individual, providing graphical representation of baseline FCM levels and the coefficient of variation (CV%) in FCM levels to be determined. For all analyses, FCM was the dependant variable. For each individual, the mean FCM concentration (FCM ± SEM), and mean CV% were calculated. Variance in the data was high, and the underlying distribution was not normal, so analysis required all data to be log-transformed. A general linear model (repeated-measures ANOVA; SPSS) tested the difference in mean FCM levels within and between tigers, investigating the effects of time and sex as factors in the model (FCM = tiger × time × sex) and significance was reported at a level of *P* ≤ 0.05. *Post hoc* multiple comparisons were made using Tukey's tests (Tukey's HSD; SPSS). Husbandry details for each individual studied at Melbourne Zoo are provided in Table 1.

Furthermore, mean FCM values and ranges of FCMs were compared with tigers from Dreamworld Themepark and Australia Zoo (Queensland, Australia: Narayan *et al.*, 2013a). Results from all three facilities were analysed using a general linear model (GLM; SPSS) to test for significance differences in FCM between zoos and sexes. Dreamworld Themepark manages one male and four female individuals of the Sumatran sub-species. Australia Zoo manages three male and three female individuals of the Sumatran sub-species.

## Results

### Laboratory EIA validation

**Figure 1:**
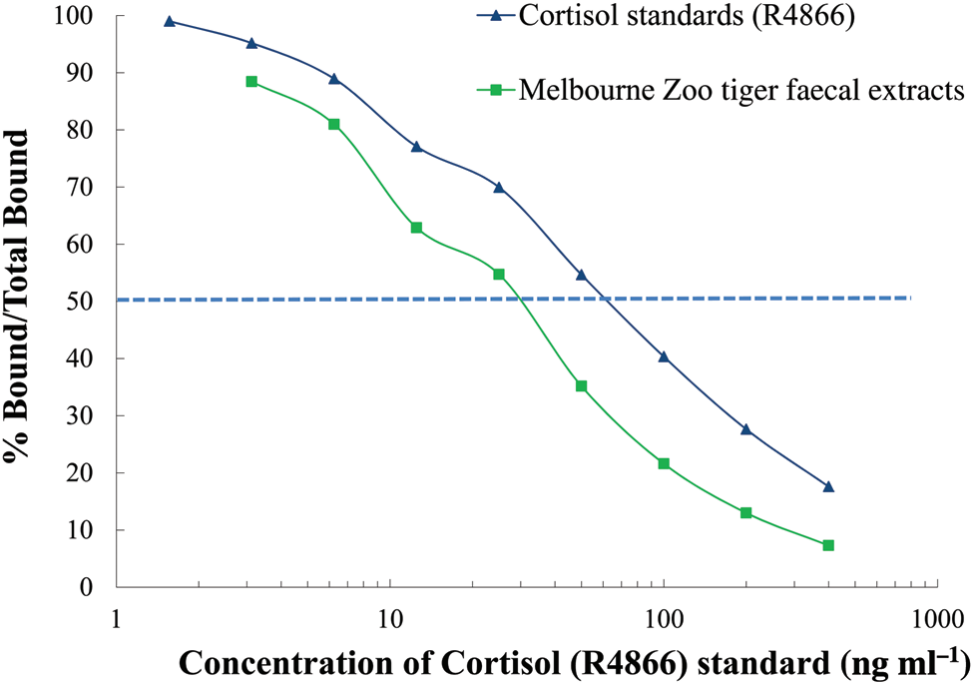
Binding displacement curves of serially diluted pooled-faecal extracts against the cortisol standard to validate the enzyme immunoassay. The *y*-axis shows the percentage of hormone bound/total binding. The 50% binding point (represented by the dashed line) determined the dilution factor (1:16) for Melbourne Zoo tiger faecal extracts.

**Figure 2:**
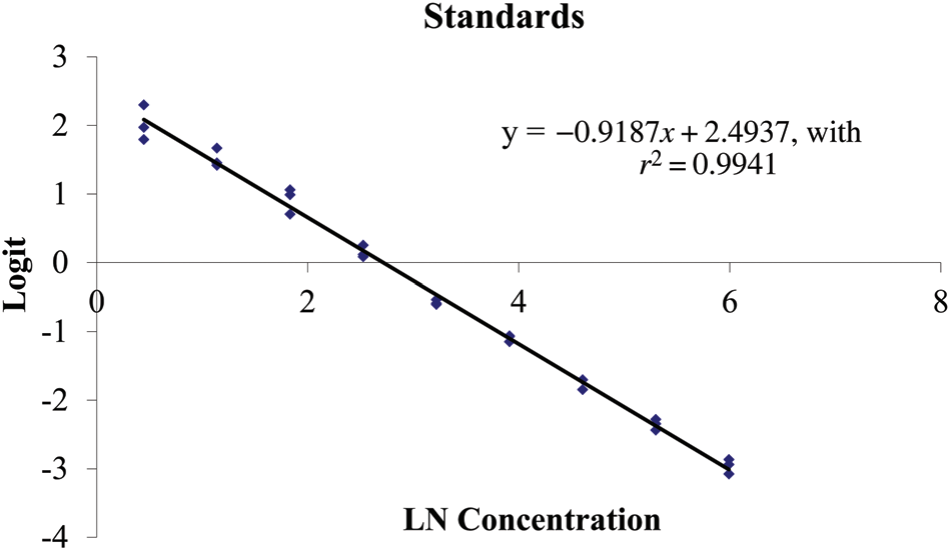
Regression plot for recovery of cortisol standard in the extract pool to achieve extraction efficiency (extraction efficiency = 99%).

Parallelism for FCMs was successfully demonstrated between the pooled tiger faecal extract against the standard cortisol R4866 curve (Fig. 1). These validation methods were also useful for detecting the 50% binding point, which determined the dilution factor (×16) for sample extracts required to run each assay (Fig. 1). Validations also achieved an extraction efficiency of >99% of cortisol hormone recovered. Recovery of cortisol, presented as a linear regression, was *y* = −0.9187*x* + 2.4937, with *r*^2^ = 0.9941 (Fig. 2); in which *y* is the concentration of hormone observed, *x* is the concentration expected, and the constant (2.49) is the *y* intercept. The sensitivity of the FCM EIA was 0.5 ± 0.1 pg per well (*n* = 50 plates analysed). The intra- and inter-assay coefficients of variation were 2.0 and 6.2%, respectively, for the high-binding internal control and 1.2 and 10.1%, respectively, for the low-binding internal control.

### Individual FCM profiles

**Figure 3:**
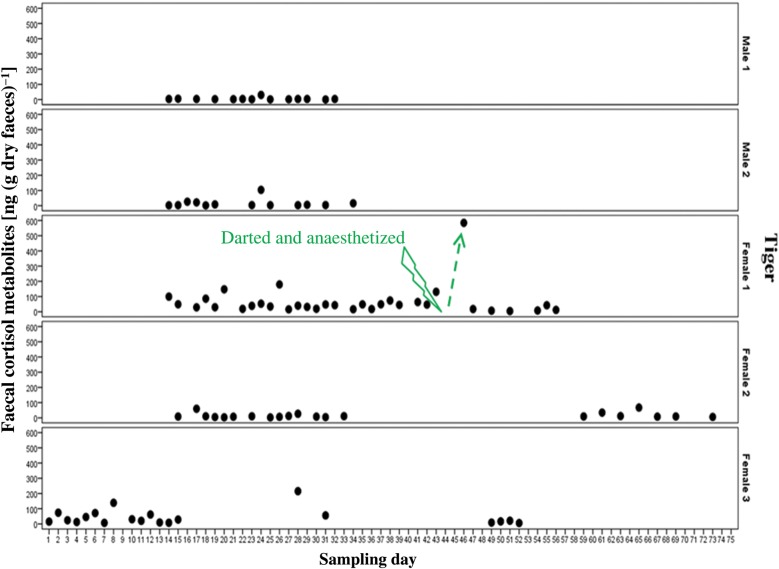
Individual faecal cortisol metabolite profiles obtained from enzyme immunoassays on faecal samples collected from tigers (*n* = 5) at Melbourne Zoo (Victoria) over a 2 month period. One data point was excluded from statistical analysis because it was attributed to the unnatural stress event of darting and anaesthesia of Female 1.

Transient variation in FCM concentrations were observed throughout the study. Male 1 had mean CV of 154%, lower than Male 2, which had the highest mean CV of 177%. Mean CVs for females were 154, 115 and 119%, respectively. An FCM response to darting and anaesthesia was detected in Female 1 on day 46 (2 days after the darting event) by a significantly elevated FCM level (584.17 ng g^−1^), >10 times higher than the baseline FCM level (48.43 ± 7.26 ng g^−1^; Fig. 3). This result is consistent with the known lag time of FCMs of ∼2–3 days following a known mild stressor, e.g. blood collection (Naidenko *et al.*, 2011, 2013a). Mean FCMs returned to baseline by day 47 (3 days after the darting event). We excluded this outlier data point from analysis of the mean baseline FCM values between sexes.

### Comparison of FCM levels between individual tigers

**Figure 4:**
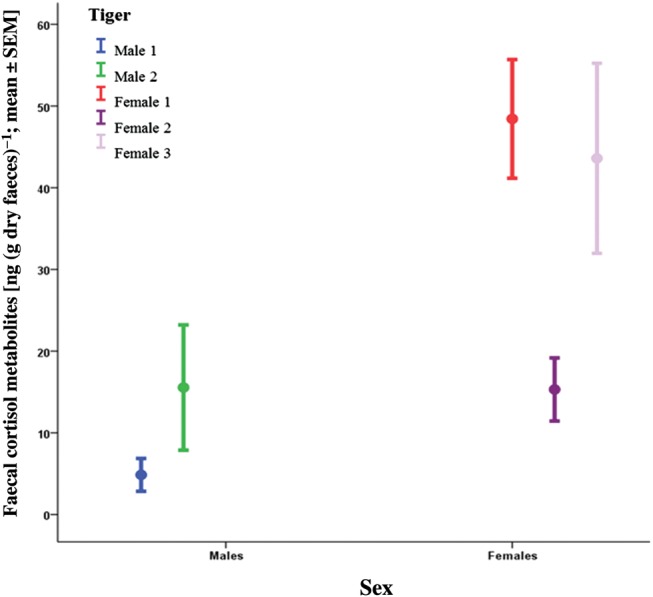
Mean faecal cortisol metabolite concentration (±1 SEM) for Melbourne Zoo's tigers (*n* = 5), clustered by sex.

Mean FCM concentration and variation (CV%) for each tiger are presented in Table 2. The FCM values across all individuals ranged from 0.24 to 215.31 ng g^−1^. Females 1 and 3 had the highest mean FCM values (48.43 ± 7.26 and 43.60 ± 11.63 ng g^−1^, respectively), with the values for Female 2 being notably lower (15.31 ± 3.87 ng g^−1^) and more similar to the means of Male 1 (4.86 ± 2.01 ng g^−1^) and Male 2 (15.55 ± 7.67 ng g^−1^; Fig. 4). The mean FCM concentration for combined males (*n* = 2) of 10.0 ± 3.9 ng g^−1^ was considerably lower than for combined females (*n* = 3), with a mean FCM value of 37.6 ± 4.9 ng g^−1^ (Fig. 4). There was a significant effect of sex and individual (GLM ANOVA: sex, *F* = 10.83, *P* < 0.001; and individual, *F* = 6.071, *P* < 0.001), but time was not significant (*F* = 0.684, *P* = 0.910). Males 1 and 2 were not significantly different from Female 2; Females 1 and 3 were not significantly different from each other, nor were Males 1 and 2 significantly different from each other (Tukey's *post hoc* multiple comparisons; *P* > 0.05).

**Table 2:** Husbandry details for tigers (*n* = 5) at Melbourne Zoo, Victoria, Australia

	Individual tiger				
	Aceh	Hutan	Indrah	Rani	Binjai
Sex	Male 1	Male 2	Female 1	Female 2	Female 3
Date of birth	9 February 2010	9 February 2010	9 February 2010	9 February 2010	30 August 2002
Body weight (kg)	113.9	113.2	83.8	81.3	87
Enclosure details	Alternated between 400 m^2^ public exhibit (with access to two 9 m^2^ dens overnight) and a 40 m^2^ off-limit area (with 9 m^2^ den access at night)				Permanently kept in 250 m^2^ public exhibit. Access to ∼6 m^2^ off-limit yard and 5 m^2^ den at night
Social arrangement	Male siblings kept together in same enclosure		Female siblings kept together in same enclosure, prior to Rani's removal and transportation to another facility		Solitary
Reproductive status	Intact, not yet used for breeding				Intact, breeding female

### Comparisons of mean FCMs of tigers between Australian zoos

**Figure 5:**
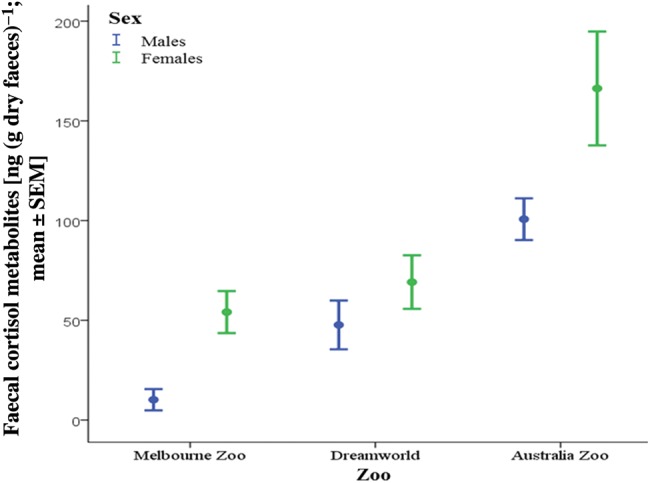
Mean faecal cortisol metabolite values (±1 SEM) for male (blue) and female tigers (green) at Melbourne Zoo (*n* = 2 males and 3 females), Dreamworld (*n* = 8 males and 5 females) and Australia Zoo (*n* = 4 males and 4 females).

The mean FCM concentration of Melbourne Zoo's tigers (*n* = 5, mean FCM = 35.6 ± 6.7 ng g^−1^) was significantly lower than that reported for the tigers at Dreamworld and Australia Zoo (GLM ANOVA; zoo and sex were significant, with *F* = 30.82, *P* < 0.001 and *F* = 8.271, *P* = 0.04, respectively; Fig. 5; Dreamworld, *n* = 13, mean FCM = 67.8 ± 4.1 ng g^−1^; and Australia Zoo, *n* = 8, mean FCM = 146.8 ± 18.1 ng g^−1^) (Narayan *et al.*, 2013a). The highest mean FCM levels were for Australia Zoo female tigers (Fig. 5).

## Discussion

We successfully quantified FCM levels in the Sumatran tigers at Melbourne Zoo using EIAs. These baseline FCM data provide valuable foundation for long-term monitoring of health and wellbeing of tigers, to allow for improvements in management and husbandry for Sumatran tigers (important members of Australia's on-going tiger captive breeding programme). These data will also be valuable for comparisons with new FCM data in the event of any illnesses, environmental modifications (such as the implementation of new enrichment programmes) or changes in social arrangement. This study, therefore, provides necessary reference FCM data for Sumatran tigers to enable the impacts and effects of potential stressors associated with captivity to be assessed quantitatively (Hill and Broom, 2009).

Comparisons of average FCM concentrations with tigers at two other Australian zoos (Narayan *et al.*, 2013a) suggest that the tigers in Australian zoos have a range of FCM levels, and future studies could refer to the reported baseline FCM data when monitoring the welfare of individuals managed in Australian zoos. Throughout the literature, there is a range of FCM values obtained from studies on closely related tiger species; however, inter-species comparisons of FCMs were limited due to the potential difference in assay systems (antibodies) used in these reports. For example, Naidenko *et al.* (2011) report a range of FCM levels between 363 and 783 ng  g^−1^ in Siberian tigers, while Young *et al.* (2004) have reported information on several other carnivore species, returning mean values of 234.1 ng g^−1^ for the domestic cat (*Felis catus*), 751.1 ng g^−1^ for the cheetah (*Acinonyx jubatus*) and 282.9 ng g^−1^ for the clouded leopard (*Neofelis nebulosa*). Physiological stress responses and the activity of the hypothalamic–pituitary–adrenal axis not only vary between closely related species but are specific to individuals of the same species (Romero, 2004), which will account for much of the variation in FCM levels found among tigers in Australian zoos. The variability in FCMs among individuals in this study most probably reflects unique intra-specific metabolic patterns, as well as each individual's capacity to adapt and cope with their environmental surroundings (Wielebnowski *et al.*, 2002a), providing useful information for keepers about the stress reactivity of tigers at an individual level.

Some degree of individual variation in FCM levels might be explained by sex differences. Female Sumatran tigers at Melbourne Zoo had a higher average mean FCM than male tigers, as reported in previous studies (Brown and Wildt, 1997; Fanson *et al.*, 2012; Narayan *et al.*, 2013a). Higher variation in FCM levels within and between females has commonly been associated with differences in stages of the reproductive cycle (Kudielka and Kirschbaum, 2005; Fanson *et al.*, 2012), including oestrous, gestation and lactation, which may have differing metabolic demands (Terio *et al.*, 2004; Pride, 2005; Kinoshita *et al*, 2011). Sex-related differences could also be due to differences in FCM excretion owing to variation in metabolic rates, excretion routes and pituitary responsiveness (Goymann *et al.*, 1999, 2001).

An acute physiological stress response was detected following the darting and anaesthesia of Female 1, in preparation for transportation to another local facility. This stressful event resulted in a significant elevation in FCM levels above baseline levels in 2 days. This lag time of 2 days for the expression of these elevated FCMs was consistent with the delay time of FCMs in response to acute stressors (such as transportation and blood sampling events) reported in earlier studies (Conforti *et al.*, 2012; Narayan *et al.*, 2013a). Such information related to the stress endocrine activity can allow zoo management to understand the effect of human interventions and find alternatives or improvements when they incur a negative impact. Although the darting and anaesthesia resulted in a significant elevation in FCMs, this response can be considered as acute (FCMs returning to baseline 3 days after the event) and should have had no direct long-term consequences for the animal's health and wellbeing.

The physiological responses of animals to external stressors can also be influenced greatly by the environment in which they live (Schwarzenberger, 2007). Previous studies on a range of other felid species have shown that stress results from novel housing conditions and lack of environmental enrichment (Wielebnowski *et al.*, 2002a; Moreira *et al.*, 2007; Szokalski *et al.*, 2012). However, the enclosures at Melbourne Zoo are spacious and naturalistic in design, with dense vegetation coverage. Furthermore, the regular addition of enrichment items offered in the enclosures also prevents boredom, stimulates the tigers and encourages active behaviours to reduce stress (Szokalski *et al.*, 2012).

Comparison of the present results with our previous study using the same protocols (Narayan *et al.*, 2013a) showed that Melbourne Zoo's tigers have slightly lower FCM averages than the tigers in two other Australian zoos. This could be related to a range of factors including differences in social group size, composition and relatedness, management approaches, enclosures characteristics, climatic differences or time of the year. For example, the Melbourne Zoo animals are related to each other, while the other two zoos have sourced animals from a variety of other facilities. Therefore, the smaller social groupings of closely related animals at Melbourne Zoo might translate to reduced social dominance or subordination that have previously been reported to result in heightened FCM levels (Creel, 2001; Goymann *et al.*, 2001; Wielebnowski *et al.*, 2002b). Alternatively, the differences between zoos may simply represent individual/genetic variation in baseline FCM levels. The degree of keeper interaction and handling of tigers also differs between these zoos but it is not possible to attribute variation in FCM levels to such management differences because of the many other differences. Moreover, while human (i.e. keeper) interaction that includes positive re-enforcement during training can increase arousal, and consequently elevated FCM levels (Szokalski *et al.* 2012), it has also been associated with increased reproductive success, reduced pacing and enhanced cognitive abilities (Mellen and Shepherdson, 1997; Swaisgood and Shepherdson, 2005).

In conclusion, continued non-invasive assessments of stress endocrinology will enable us to evaluate the effectiveness of enrichment strategies for tigers and improving wellbeing in captivity through such management approaches. The influence of many factors, such as diet, seasonal rhythms, sample storage and preservation techniques, reproductive status, social arrangements and exposure to visitors are widely discussed in published literature but are yet to be explored rigorously (Sapolsky *et al.*, 2000; Goymann *et al.*, 2001; Millspaugh and Washburn, 2004). These effects could all contribute to variation when interpreting results to gain meaningful insight into the tiger's stress endocrine function. With these considerations, the use of FCMs to monitor stress in tigers has the potential to act as a powerful biomarker for the physiological effects of the captive environment on the health and welfare of these charismatic big cats.
